# Supplementary material for: Time-series transcriptome analysis identified differentially expressed genes in broiler chicken infected with mixed Eimeria species
Source: Front Genet. 2022 Aug 8;13:886781. doi: 10.3389/fgene.2022.886781 (PMC9393255; doi:10.3389/fgene.2022.886781)
Supplement: Supplementary file 2 [file DataSheet1.ZIP › 7dpi_GO.Gsea.1625106495923/GOCC_RESPIRASOME.html]

Details for gene set GOCC\_RESPIRASOME[GSEA]

|  || Dataset | TMM\_7dpi\_gct\_format\_7dpi\_gct\_format.Class\_4dpi.cls #PC\_versus\_NC.Class\_4dpi.cls #PC\_versus\_NC\_repos |
| Phenotype | Class\_4dpi.cls#PC\_versus\_NC\_repos |
| Upregulated in class | 1 |
| GeneSet | GOCC\_RESPIRASOME |
| Enrichment Score (ES) | 0.78255844 |
| Normalized Enrichment Score (NES) | 3.6673615 |
| Nominal p-value | 0.0 |
| FDR q-value | 0.0 |
| FWER p-Value | 0.0 |
Table: GSEA Results Summary

  

Fig 1: Enrichment plot: GOCC\_RESPIRASOME      
 Profile of the Running ES Score & Positions of GeneSet Members on the Rank Ordered List

  

| SYMBOL | TITLE | RANK IN GENE LIST | RANK METRIC SCORE | RUNNING ES | CORE ENRICHMENT || 1 | HIGD2A | na | 210 | 1.029 | 0.0130 | Yes |
| 2 | COX5A | na | 300 | 0.942 | 0.0334 | Yes |
| 3 | NDUFA2 | na | 303 | 0.935 | 0.0610 | Yes |
| 4 | NDUFB6 | na | 307 | 0.933 | 0.0884 | Yes |
| 5 | NDUFS5 | na | 308 | 0.932 | 0.1160 | Yes |
| 6 | NDUFB7 | na | 323 | 0.915 | 0.1420 | Yes |
| 7 | NDUFB2 | na | 348 | 0.885 | 0.1662 | Yes |
| 8 | NDUFAB1 | na | 356 | 0.876 | 0.1916 | Yes |
| 9 | NDUFA7 | na | 371 | 0.857 | 0.2159 | Yes |
| 10 | NDUFC2 | na | 394 | 0.834 | 0.2387 | Yes |
| 11 | UQCR10 | na | 444 | 0.785 | 0.2579 | Yes |
| 12 | CYCS | na | 475 | 0.763 | 0.2780 | Yes |
| 13 | COX6A1 | na | 480 | 0.760 | 0.3002 | Yes |
| 14 | UQCR11 | na | 485 | 0.754 | 0.3223 | Yes |
| 15 | UQCRB | na | 487 | 0.753 | 0.3445 | Yes |
| 16 | NDUFA11 | na | 497 | 0.746 | 0.3659 | Yes |
| 17 | COX7A2 | na | 499 | 0.743 | 0.3878 | Yes |
| 18 | SDHAF4 | na | 516 | 0.730 | 0.4081 | Yes |
| 19 | NDUFA13 | na | 557 | 0.708 | 0.4258 | Yes |
| 20 | NDUFS3 | na | 623 | 0.675 | 0.4403 | Yes |
| 21 | NDUFB3 | na | 656 | 0.657 | 0.4571 | Yes |
| 22 | NDUFS8 | na | 661 | 0.653 | 0.4762 | Yes |
| 23 | NDUFS6 | na | 686 | 0.642 | 0.4932 | Yes |
| 24 | SDHD | na | 708 | 0.632 | 0.5102 | Yes |
| 25 | UQCC3 | na | 735 | 0.620 | 0.5264 | Yes |
| 26 | NDUFA1 | na | 742 | 0.616 | 0.5442 | Yes |
| 27 | UQCRHL | na | 788 | 0.594 | 0.5580 | Yes |
| 28 | NDUFA5 | na | 794 | 0.593 | 0.5752 | Yes |
| 29 | SDHB | na | 840 | 0.575 | 0.5885 | Yes |
| 30 | NDUFB5 | na | 902 | 0.554 | 0.5998 | Yes |
| 31 | NDUFV3 | na | 988 | 0.527 | 0.6083 | Yes |
| 32 | NDUFB4 | na | 1015 | 0.517 | 0.6214 | Yes |
| 33 | NDUFA12 | na | 1023 | 0.513 | 0.6361 | Yes |
| 34 | COX7B | na | 1055 | 0.501 | 0.6483 | Yes |
| 35 | NDUFA6 | na | 1079 | 0.492 | 0.6610 | Yes |
| 36 | NDUFA8 | na | 1080 | 0.492 | 0.6756 | Yes |
| 37 | UQCRQ | na | 1103 | 0.484 | 0.6881 | Yes |
| 38 | NDUFS2 | na | 1124 | 0.477 | 0.7006 | Yes |
| 39 | COX4I1 | na | 1125 | 0.476 | 0.7147 | Yes |
| 40 | SDHC | na | 1135 | 0.473 | 0.7280 | Yes |
| 41 | NDUFB10 | na | 1215 | 0.450 | 0.7347 | Yes |
| 42 | UQCRFS1 | na | 1263 | 0.437 | 0.7437 | Yes |
| 43 | NDUFV1 | na | 1307 | 0.428 | 0.7528 | Yes |
| 44 | NDUFB8 | na | 1330 | 0.423 | 0.7635 | Yes |
| 45 | NDUFS7 | na | 1371 | 0.412 | 0.7724 | Yes |
| 46 | NDUFV2 | na | 1395 | 0.408 | 0.7826 | Yes |
| 47 | UQCRC2 | na | 1700 | 0.332 | 0.7669 | No |
| 48 | COX7A2L | na | 1761 | 0.316 | 0.7713 | No |
| 49 | NDUFB9 | na | 1842 | 0.299 | 0.7735 | No |
| 50 | COX15 | na | 1927 | 0.282 | 0.7748 | No |
| 51 | NDUFB1 | na | 1995 | 0.272 | 0.7773 | No |
| 52 | BCS1L | na | 2225 | 0.232 | 0.7650 | No |
| 53 | UQCRC1 | na | 2326 | 0.217 | 0.7631 | No |
| 54 | NDUFA4 | na | 2726 | 0.160 | 0.7344 | No |
| 55 | NDUFA9 | na | 2879 | 0.141 | 0.7259 | No |
| 56 | NDUFA10 | na | 3412 | 0.073 | 0.6835 | No |
| 57 | SDHA | na | 3488 | 0.064 | 0.6791 | No |
| 58 | NDUFS1 | na | 3763 | 0.033 | 0.6571 | No |
| 59 | NDUFAF1 | na | 3921 | 0.014 | 0.6444 | No |
| 60 | SURF1 | na | 3926 | 0.013 | 0.6444 | No |
| 61 | NDUFS4 | na | 4402 | -0.040 | 0.6059 | No |
| 62 | TTC19 | na | 4743 | -0.075 | 0.5796 | No |
| 63 | COX7C | na | 4881 | -0.090 | 0.5709 | No |
| 64 | FOXRED1 | na | 6248 | -0.229 | 0.4633 | No |
| 65 | NNT | na | 9557 | -0.639 | 0.2053 | No |
Table: GSEA details [plain text format]

  

Fig 2: GOCC\_RESPIRASOME      
 Blue-Pink O' Gram in the Space of the Analyzed GeneSet

  

Fig 3: GOCC\_RESPIRASOME: Random ES distribution      
 Gene set null distribution of ES for **GOCC\_RESPIRASOME**
